# Comparative effectiveness of nirmatrelvir/ritonavir versus sotrovimab and molnupiravir for preventing severe COVID-19 outcomes in non-hospitalised high-risk patients during Omicron waves: observational cohort study using the OpenSAFELY platform

**DOI:** 10.1016/j.lanepe.2023.100741

**Published:** 2023-10-08

**Authors:** Bang Zheng, John Tazare, Linda Nab, Amelia CA. Green, Helen J. Curtis, Viyaasan Mahalingasivam, Emily L. Herrett, Ruth E. Costello, Rosalind M. Eggo, Victoria Speed, Sebastian CJ. Bacon, Christopher Bates, John Parry, Jonathan Cockburn, Frank Hester, Sam Harper, Andrea L. Schaffer, William J. Hulme, Amir Mehrkar, Stephen JW. Evans, Brian MacKenna, Ben Goldacre, Ian J. Douglas, Laurie A. Tomlinson, the OpenSAFELY Collaborative

**Affiliations:** a London School of Hygiene and Tropical Medicine, Keppel Street, London WC1E 7HT, UK; b Nuffield Department of Primary Care Health Sciences, Bennett Institute for Applied Data Science, University of Oxford, Oxford OX2 6GG, UK; c TPP, TPP House, 129 Low Lane, Horsforth, Leeds LS18 5PX, UK

**Keywords:** COVID-19, Comparative effectiveness, Paxlovid, Sotrovimab, Real-world data

## Abstract

**Background:**

Timely evidence of the comparative effectiveness between COVID-19 therapies in real-world settings is needed to inform clinical care. This study aimed to compare the effectiveness of nirmatrelvir/ritonavir versus sotrovimab and molnupiravir in preventing severe COVID-19 outcomes in non-hospitalised high-risk COVID-19 adult patients during Omicron waves.

**Methods:**

With the approval of NHS England, we conducted a real-world cohort study using the OpenSAFELY-TPP platform. Patient-level primary care data were obtained from 24 million people in England and were securely linked with data on COVID-19 infection and therapeutics, hospital admission, and death, covering a period where both nirmatrelvir/ritonavir and sotrovimab were first-line treatment options in community settings (February 10, 2022–November 27, 2022). Molnupiravir (third-line option) was used as an exploratory comparator to nirmatrelvir/ritonavir, both of which were antivirals. Cox proportional hazards model stratified by area was used to compare the risk of 28-day COVID-19 related hospitalisation/death across treatment groups.

**Findings:**

A total of 9026 eligible patients treated with nirmatrelvir/ritonavir (n = 5704) and sotrovimab (n = 3322) were included in the main analysis. The mean age was 52.7 (SD = 14.9) years and 93% (8436/9026) had three or more COVID-19 vaccinations. Within 28 days after treatment initiation, 55/9026 (0.61%) COVID-19 related hospitalisations/deaths were observed (34/5704 [0.60%] treated with nirmatrelvir/ritonavir and 21/3322 [0.63%] with sotrovimab). After adjusting for demographics, high-risk cohort categories, vaccination status, calendar time, body mass index and other comorbidities, we observed no significant difference in outcome risk between nirmatrelvir/ritonavir and sotrovimab users (HR = 0.89, 95% CI: 0.48–1.63; P = 0.698). Results from propensity score weighted model also showed non-significant difference between treatment groups (HR = 0.82, 95% CI: 0.45–1.52; P = 0.535). The exploratory analysis comparing nirmatrelvir/ritonavir users with 1041 molnupiravir users (13/1041 [1.25%] COVID-19 related hospitalisations/deaths) showed an association in favour of nirmatrelvir/ritonavir (HR = 0.45, 95% CI: 0.22–0.94; P = 0.033).

**Interpretation:**

In routine care of non-hospitalised high-risk adult patients with COVID-19 in England, no substantial difference in the risk of severe COVID-19 outcomes was observed between those who received nirmatrelvir/ritonavir and sotrovimab between February and November 2022, when Omicron subvariants BA.2, BA.5, or BQ.1 were dominant.

**Funding:**

10.13039/100014013UK Research and Innovation, 10.13039/100010269Wellcome Trust, 10.13039/501100000265UK Medical Research Council, 10.13039/501100000272National Institute for Health and Care Research, and 10.13039/501100023699Health Data Research UK.

**Research in context**

**Evidence before this study**

We searched PubMed with the terms ((nirmatrelvir OR Paxlovid OR Sotrovimab OR Xevudy) AND (COVID-19 OR SARS-CoV-2) AND outcome) for relevant studies published in English by March 31, 2023. The following two randomised controlled trials (RCT) in unvaccinated patients before the Omicron wave showed efficacy of nirmatrelvir/ritonavir and sotrovimab compared to placebo. The EPIC-HR trial was a phase 2/3 double-blind RCT for nirmatrelvir/ritonavir in symptomatic, non-hospitalised adults at high risk for progression to severe COVID-19. In the final analysis of 1379 patients there was a reduced risk of COVID-19 related hospitalisation or all-cause death within 28 days in the nirmatrelvir/ritonavir group compared with the placebo group (0.72% versus 6.53%; relative risk = 0.11; P < 0.001). The COMET-ICE trial was a phase 3 double-blind RCT that evaluated the use of intravenous sotrovimab in non-hospitalised high-risk adult patients with symptomatic COVID-19. In the final sample of 1057 patients, treatment group had a reduced risk of all-cause hospitalisation or death within 29 days compared with the placebo group (1% versus 6%; adjusted relative risk = 0.21; P < 0.001). However, no comparative trials on these two medications or other COVID-19 therapies have been conducted. As for the observational data, several real-world studies in non-hospitalised patients (mostly during the Omicron BA.1/BA.2 era) have shown clinical effectiveness of nirmatrelvir/ritonavir compared with non-users, while the real-world evidence on sotrovimab was inconsistent. Comprehensive comparative effectiveness analysis across these treatment options with large-scale observational data remains limited, especially after the Omicron BA.2 wave.

**Added value of this study**

Based on the OpenSAFELY-TPP platform with electronic health record data from ∼40% of the population of England, we conducted an observational study on the comparative effectiveness of nirmatrelvir/ritonavir versus alternative antiviral medications among non-hospitalised high-risk COVID-19 patients, covering the Omicron BA.2, BA.5 and BQ.1-dominant periods. Our analysis with multisource real-world data did not show significant difference in the risk of COVID-19 related hospitalisation/death between nirmatrelvir/ritonavir and sotrovimab users. In contrast, the exploratory analyses with molnupiravir users as the comparison group showed a significantly lower risk of severe COVID-19 outcomes among patients prescribed nirmatrelvir/ritonavir. To our knowledge, this is one of the first large real-world comparative effectiveness studies during the recent Omicron waves, with granular data that enabled extensive adjustments for confounding and exclusions for contraindications and drug interactions for the use of nirmatrelvir/ritonavir.

**Implications of all the available evidence**

In routine care of non-hospitalised high-risk adult patients with COVID-19 in England, no substantial difference in the risk of severe COVID-19 outcomes was observed between those who received nirmatrelvir/ritonavir and sotrovimab across Omicron BA.2, BA.5, and BQ.1-dominant periods, during which both medications were first-line treatment options in community settings.

## Introduction

On December 16, 2021, COVID-19 Medicine Delivery Units (CMDUs) were launched across England to provide antiviral medicines and neutralising monoclonal antibodies (nMAbs) to treat symptomatic COVID-19 patients at high risk of severe outcomes in community settings. The clinical guidance from National Health Service (NHS) England^1^^,^^2^ has been revised over time based on emerging evidence and the approval of new medications by the UK Medicines and Healthcare products Regulatory Agency (MHRA). After February 10, 2022, nirmatrelvir/ritonavir (Paxlovid, an oral antiviral) and sotrovimab (an intravenous nMAb) were both recommended as first-line treatments for non-hospitalised high-risk COVID-19 patients to prevent disease progression.^2^

The approval and early routine clinical use of these two medications were mainly supported by evidence from two randomised controlled trials (RCT) in unvaccinated population before the Omicron wave,^3^^,^^4^ in which certain clinically vulnerable subgroups, such as immunosuppressed individuals, were underrepresented. Several real-world observational studies in non-hospitalised patients during the Omicron era have shown effectiveness of nirmatrelvir/ritonavir compared with non-users.^5^^,^^6^ However, substantial confounding in these analyses was possible given differences in the characteristics and clinical conditions between treated and untreated patients (e.g., untreated patients could have been at low-risk of severe outcomes, or at high-risk but with contraindications to nirmatrelvir/ritonavir^7^). Comparative effectiveness analysis for treatments prescribed under similar clinical indications, with careful consideration of the contraindications and drug interactions for the use of nirmatrelvir/ritonavir,^2^ should increase comparability of participants, leading to more robust findings. Comparative evidence is also useful in clinical settings where the decision is over which treatment to use rather than whether to treat or not.

There has been uncertainty about the real-world effectiveness of sotrovimab due to its reduced activity against several Omicron variants in in vitro studies, and an updated World Health Organization (WHO) guideline makes a strong recommendation against use of sotrovimab for the treatment of non-severe COVID-19 patients.^8^ However, the guideline from the UK National Institute for Health and Care Excellence (NICE) published earlier this year recommends sotrovimab as a COVID-19 therapeutic option based on more recent clinical and in vitro data.^9^ In contrast, nirmatrelvir/ritonavir was strongly recommended in both guidelines, though with multiple contraindications and potential drug interactions.^8^^,^^9^ In this context, evidence of the comparative effectiveness between these two medications was urgently needed to inform clinical care.

Therefore, we conducted a real-world observational study to compare the effectiveness of nirmatrelvir/ritonavir and sotrovimab on preventing severe outcomes in non-hospitalised high-risk COVID-19 patients across England, utilising the near real-time electronic health record (EHR) data in the OpenSAFELY-TPP platform.^7^ The study period covers Omicron BA.2, BA.5 and BQ.1 waves in England. As an exploratory analysis, we also compared the risks of severe outcomes between nirmatrelvir/ritonavir users and those treated with molnupiravir^10^ (a third-line antiviral included in NHS England guidance during the study period).

## Methods

### Data source

OpenSAFELY is a data analytics platform created by our team on behalf of NHS England to address urgent COVID-19 research questions (https://opensafely.org). The near real-time data analysed within OpenSAFELY is based on 24 million people currently registered with General Practice (GP) surgeries using TPP SystmOne software. It includes pseudonymised data such as coded diagnoses, medications and physiological parameters. Patient-level vaccination status is available in the GP records directly via the National Immunisation Management System (NIMS). No free text data are included. The following linked data were also used for this study: Office for National Statistics (ONS) mortality records; in-patient hospital spell records via Secondary Uses Service (SUS); national coronavirus testing records via the Second Generation Surveillance System (SGSS); and the “COVID-19 therapeutics dataset”, a patient-level dataset on antiviral and nMAbs treatments, newly sourced from NHS England, derived from Blueteq software that CMDUs use to notify NHS England of COVID-19 treatments.^7^

### Study design and population

We conducted a cohort study of all adults (≥18 years old) within OpenSAFELY-TPP who had treatment records of either nirmatrelvir/ritonavir, sotrovimab or molnupiravir from CMDUs between February 10, 2022 and November 27, 2022, after which sotrovimab was de-prioritised in the NHS England guidance and its use was only to be considered by exception where other antivirals were contraindicated or unsuitable.^11^ In addition, eligible patients in this study were required to be non-hospitalised for COVID-19 at treatment initiation (as recorded in the COVID-19 therapeutics dataset^7^), and be registered in GP surgeries before treatment. Patients were excluded if they had treatment records of any other antivirals or nMAbs for COVID-19 before receiving the treatment under investigation (n = 94). Patients with treatment records of other antivirals or nMAbs after receiving the treatment under investigation were censored at the start date of that second treatment (n = 39).

**Fig. 1:**
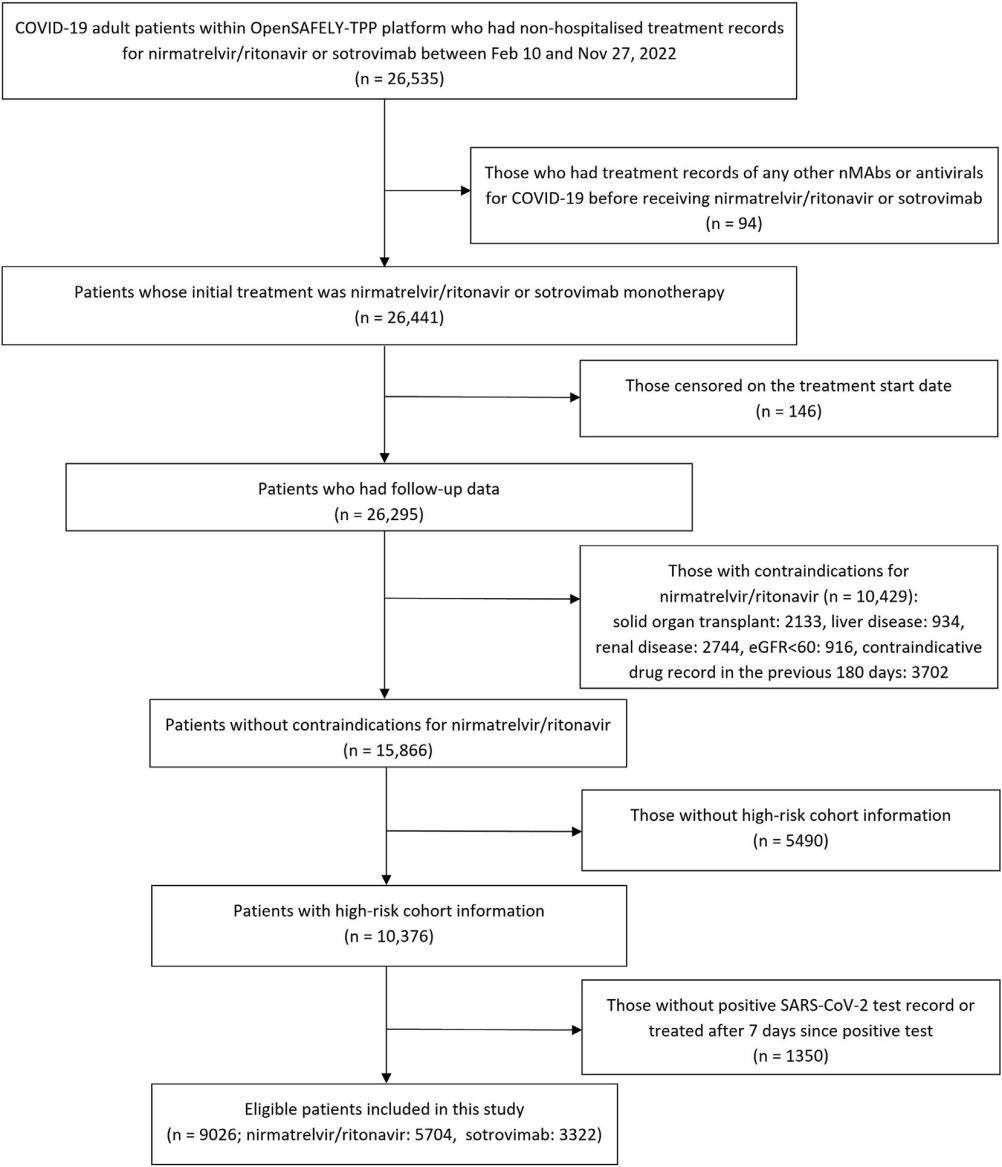
Flowchart for study participants.

According to the eligibility criteria from NHS England,^2^ patients needed to have SARS-CoV-2 infection confirmed by polymerase chain reaction (PCR) testing or lateral flow test, onset of COVID-19 symptoms within the last five days (or within seven days if clinically indicated), and be a member of at least one of the following ten high-risk cohorts (determined by the Department of Health and Social Care commissioned Independent Advisory Group): patients with Down syndrome, a solid cancer, a haematological disease or stem cell transplant, renal disease, liver disease, immune-mediated inflammatory disorders (e.g., rheumatoid arthritis, lupus), immune deficiencies, HIV/AIDS, solid organ transplant, or rare neurological conditions. Therefore, we excluded those who initiated treatment more than seven days after a positive test date or had no positive test record, and those who were not classified as high-risk cohort member based on their health records (Fig. 1).

To be noted, based on the NHS England guidance^2^ patients were ineligible to receive nirmatrelvir/ritonavir if they had a history of advanced decompensated liver cirrhosis, stage 3–5 chronic kidney disease (or stage 4–5 in the updated guidance), solid organ transplant, or were taking any of the contraindicated medications listed as ‘do not use’ in the Specialist Pharmacy Service (SPS) guidance.^12^ Therefore, to maximise comparability between patients administered nirmatrelvir/ritonavir versus sotrovimab or molnupiravir, we excluded all patients with these restrictions based on diagnosis codes, clinical tests (eGFR or creatinine for kidney disease) and medication records (prescription of any contraindicated medications in “do not use” codelist^12^ within 180 days before treatment) (Fig. 1).

### Study measures

#### Exposure

The exposure of interest was treatment with nirmatrelvir/ritonavir versus sotrovimab administered by CMDUs in the main study, and treatment with nirmatrelvir/ritonavir versus molnupiravir in the exploratory analysis. Exposure status and date of treatment of each patient were ascertained from the COVID-19 therapeutics dataset.

#### Outcome

The primary outcome was COVID-19 related hospitalisation (based on primary diagnosis ascertained from SUS) or COVID-19 related death (based on underlying/contributing causes of death from ONS) within 28 days after treatment initiation. Secondary outcomes were 28-day all-cause hospital admission or death, and 60-day COVID-19 related hospitalisation/death. To exclude events where patients were admitted in order to receive sotrovimab infusion or other planned/regular treatment, we did not count admissions coded as “elective day case admission” or “regular admission” in SUS or day cases detected by the same admission and discharge dates as hospitalisation events for all patients.

#### Covariates

The following potential confounding factors were extracted on or before the date of treatment initiation: age, sex, Sustainability Transformation Partnerships code (an NHS administrative region), ethnicity, Index of Multiple Deprivation (IMD, by quintile derived from the patient’s postcode at lower super output area level), rural-urban classification (derived from patient’s postcode), calendar time (to account for secular trend of prescription and COVID-19 outcomes), COVID-19 vaccination status (unvaccinated, one vaccination, two vaccinations, three vaccinations, or four or more), positive test date for SARS-CoV-2 infection (PCR or lateral flow test), body mass index (BMI, most recent record within 10 years), high-risk cohort categories as mentioned above (allowing multiple categories per patient), other comorbidities (diabetes, hypertension, chronic heart diseases, chronic respiratory diseases, learning disabilities, severe mental illness), and care home residency and housebound status. Individuals with missing ethnicity, IMD and BMI were included as “Unknown” category.

### Statistical analyses

For the comparative effectiveness analysis of nirmatrelvir/ritonavir versus sotrovimab, distributions of baseline characteristics were compared between patients in these two treatment groups. Follow-up time of individual patients was calculated from the date of the treatment initiation record, until the earliest of: date of outcome event, 28 days after treatment initiation, initiation of a second nMAb/antiviral treatment, death, patient de-registration date, or the study end date (January 1, 2023).

Risks of 28-day COVID-19 related hospitalisation/death were compared between the two drug groups using Cox proportional hazards models, with time since treatment initiation as the time scale. The Cox models were stratified by area to account for geographic heterogeneity in baseline hazards, with sequential adjustment for other baseline covariates. Model 1 was adjusted for age and sex; Model 2 additionally adjusted for high-risk cohort categories (Down syndrome, solid cancer, haematological disease, immune-mediated inflammatory disorders, immunosuppression, rare neurological conditions); Model 3 further adjusted for ethnicity (White or non-White), IMD quintiles, vaccination status, calendar date (with restricted cubic splines to account for non-linear effect); and Model 4 additionally adjusted for BMI category (<25 kg/m^2^, 25–<30 kg/m^2^, ≥30 kg/m^2^), diabetes, hypertension, chronic cardiac and respiratory diseases. The proportional hazards assumption was assessed by testing for a zero slope in the scaled Schoenfeld residuals for each Cox model.

As an alternative approach to account for confounding bias, we used the propensity score weighting (PSW) method to balance the distributions of relevant covariates between groups. The propensity score (PS) for each patient is defined as the conditional probability of being treated with nirmatrelvir/ritonavir, estimated with a binary logistic regression of the received treatment (nirmatrelvir/ritonavir versus sotrovimab) on relevant baseline covariates (different set of covariates were used for Models 1–4 as mentioned above). The average treatment effect (ATE) weighting scheme based on propensity scores (with and without trimming: approaches discussed further in results^13^^,^^14^) was then applied to the Cox model. Balance check of baseline covariates after weighting was conducted using standardised mean difference (SMD) between groups. Robust variance estimators were used in the weighted Cox model. Similar analyses were conducted for secondary outcomes.

Further exploratory analyses were conducted by different subgroups, including time period with different dominant variants (February 10–May 31 for BA.2, June 1–November 27 for BA.5/BQ.1^15^), each high-risk cohort, presence of obesity (≥30 versus <30 kg/m^2^), diabetes, hypertension, chronic cardiac diseases or chronic respiratory diseases, days between test positive and treatment initiation (<3 versus 3–5 days), age group (<60 versus ≥60 years), sex and ethnicity (White versus non-White). Effect modification by each covariate was tested by adding the corresponding interaction term in the stratified Cox model, with Bonferroni correction for multiple testing.

Several sensitivity analyses based on the stratified Cox model were conducted to assess the robustness of main findings, including (1) using complete-case analysis or Multiple Imputation by Chained Equations to deal with missing values in covariates; (2) additionally adjusting for time between test positive and treatment initiation, and time between last vaccination date and treatment initiation; (3) additionally adjusting for rural-urban classification, and other comorbidities and factors that might have influenced clinician’s choice of therapy through the patient’s ability to travel to hospital for an infusion (learning disabilities, severe mental illness, care home residency or housebound status); (4) using restricted cubic splines for age to control for potential non-linear age effect; (5) excluding patients with treatment records of both sotrovimab and nirmatrelvir/ritonavir, or any other treatments during follow-up (i.e., casirivimab, molnupiravir, or remdesivir); (6) excluding patients who initiated treatment after 5 days since positive SARS-CoV-2 test; (7) adding back patients who did not have a positive test record before treatment or initiated treatment after 7 days since positive SARS-CoV-2 test; (8) adding back patients who had missing high-risk cohort information; (9) creating a 1-day or 2-day lag in the follow-up start date to account for potential delays in drug administration; (10) further excluding those with prescription records of any medications with potential interactions with nirmatrelvir/ritonavir (i.e., in “Drugs consider risks and benefits” codelist)^12^ within 180 days before treatment of nirmatrelvir/ritonavir or sotrovimab; (11) not excluding those with contraindications or “caution” medications for nirmatrelvir/ritonavir but adjusting for these conditions as covariates instead; (12) additionally adjusting for vaccine type for the latest COVID-19 vaccination, and then restricting the analysis to patients receiving the same type of vaccine; and (13) expanding the follow-up period to 90 days.

Finally, we adopted similar analytical approaches for the exploratory analysis comparing nirmatrelvir/ritonavir users with molnupiravir users. Data management was performed using Python, with analysis carried out using Stata 16.1.

### Role of the funding source

The funder of the study had no role in study design, data collection, data analysis, data interpretation, or writing of the report.

## Results

### Patient characteristics

Between February 10 and November 27, 2022, a total of 9026 non-hospitalised COVID-19 patients treated with nirmatrelvir/ritonavir (n = 5704) or sotrovimab (n = 3322) were included in the main analysis. The mean age of these patients was 52.7 (SD = 14.9) years; 67% were female, 94% White and 93% had three or more COVID-19 vaccinations. Compared with the sotrovimab group, those receiving nirmatrelvir/ritonavir were slightly younger (52.1 versus 53.8 years), had a slightly higher proportion of Down syndrome, immune-mediated inflammatory disorders and rare neurological conditions, and lower proportion of solid cancer, haematological disease, diabetes, hypertension, chronic heart diseases and chronic respiratory diseases (Table 1). There were also some geographic variations in the prescription of these two medications and greater use of sotrovimab earlier during the study period. As shown in Supplementary Fig. S1, the prescription count of sotrovimab was similar to that of nirmatrelvir/ritonavir in March 2022, but reduced to below half of the nirmatrelvir/ritonavir prescription count after July 2022. Other baseline characteristics were similar between the two groups (Table 1).

**Table 1:** Baseline characteristics of patients receiving nirmatrelvir/ritonavir or sotrovimab.

Characteristics	Nirmatrelvir/ritonavir group	Sotrovimab group	Total
N	5704	3322	9026
Age (year), mean (SD)	52.1 (14.7)	53.8 (15.2)	52.7 (14.9)
Female, n (%)	3830 (67.2)	2237 (67.3)	6067 (67.2)
White, n (%)	5245 (93.5)	3076 (93.7)	8321 (93.6)
IMD quintile, n (%)			
1 (most deprived)	585 (10.6)	350 (10.8)	935 (10.7)
2	860 (15.6)	551 (17.0)	1411 (16.1)
3	1240 (22.4)	815 (25.2)	2055 (23.5)
4	1401 (25.3)	746 (23.1)	2147 (24.5)
5 (least deprived)	1445 (26.1)	772 (23.9)	2217 (25.3)
Region (NHS), n (%)			
East	1456 (25.5)	990 (29.8)	2446 (27.1)
London	270 (4.7)	207 (6.2)	477 (5.3)
East Midlands	1345 (23.6)	749 (22.6)	2094 (23.2)
West Midlands	71 (1.2)	176 (5.3)	247 (2.7)
North East	144 (2.5)	148 (4.5)	292 (3.2)
North West	545 (9.6)	236 (7.1)	781 (8.7)
South East	542 (9.5)	148 (4.5)	690 (7.6)
South West	593 (10.4)	496 (14.9)	1089 (12.1)
Yorkshire	738 (12.9)	172 (5.2)	910 (10.1)
High risk cohorts, n (%)			
Down syndrome	244 (4.3)	100 (3.0)	344 (3.8)
Solid cancer	615 (10.8)	466 (14.0)	1081 (12.0)
Haematological disease	717 (12.6)	627 (18.9)	1344 (14.9)
Immune-mediated inflammatory diseases (e.g., rheumatoid arthritis, lupus)	2370 (41.6)	1284 (38.7)	3654 (40.5)
Immunosuppression	610 (10.7)	342 (10.3)	952 (10.6)
HIV/AIDS	17 (0.3)	13 (0.4)	30 (0.3)
Rare neurological disease	1595 (28.0)	794 (23.9)	2389 (26.5)
BMI (kg/m^2^), mean (SD)	28.3 (6.7)	28.5 (6.7)	28.4 (6.7)
Comorbidities, n (%)			
Diabetes	689 (12.1)	479 (14.4)	1168 (12.9)
Chronic cardiac disease	309 (5.4)	241 (7.3)	550 (6.1)
Hypertension	1274 (22.3)	983 (29.6)	2257 (25.0)
Chronic respiratory disease	894 (15.7)	683 (20.6)	1577 (17.5)
Vaccination status, n (%)			
None	81 (1.4)	45 (1.4)	126 (1.4)
One vaccination	77 (1.4)	33 (1.0)	110 (1.2)
Two vaccinations	223 (3.9)	131 (3.9)	354 (3.9)
Three vaccinations	2782 (48.8)	1628 (49.0)	4410 (48.9)
Four or more	2541 (44.6)	1485 (44.7)	4026 (44.6)
Days between test positive and treatment, median (IQR)	1 (1–2)	2 (1–3)	2 (1–2)
Weeks between campaign start and treatment, median (IQR)	26 (16–32)	18 (13–30)	22 (15–31)

Note: IMD, BMI, and ethnicity had 261, 874 and 131 missing values, respectively.

### Comparative effectiveness of nirmatrelvir/ritonavir versus sotrovimab for the outcome events

During the 28 days of follow-up after treatment initiation, 55 cases (0.61%) of COVID-19 related hospitalisations/deaths were observed, with 34 (0.60%) in the nirmatrelvir/ritonavir group and 21 (0.63%) in the sotrovimab group. Kaplan–Meier curves are shown in Supplementary Fig. S2. The number of COVID-19 related deaths was 9 in the nirmatrelvir/ritonavir group and ≤5 in the sotrovimab group.

**Fig. 2:**
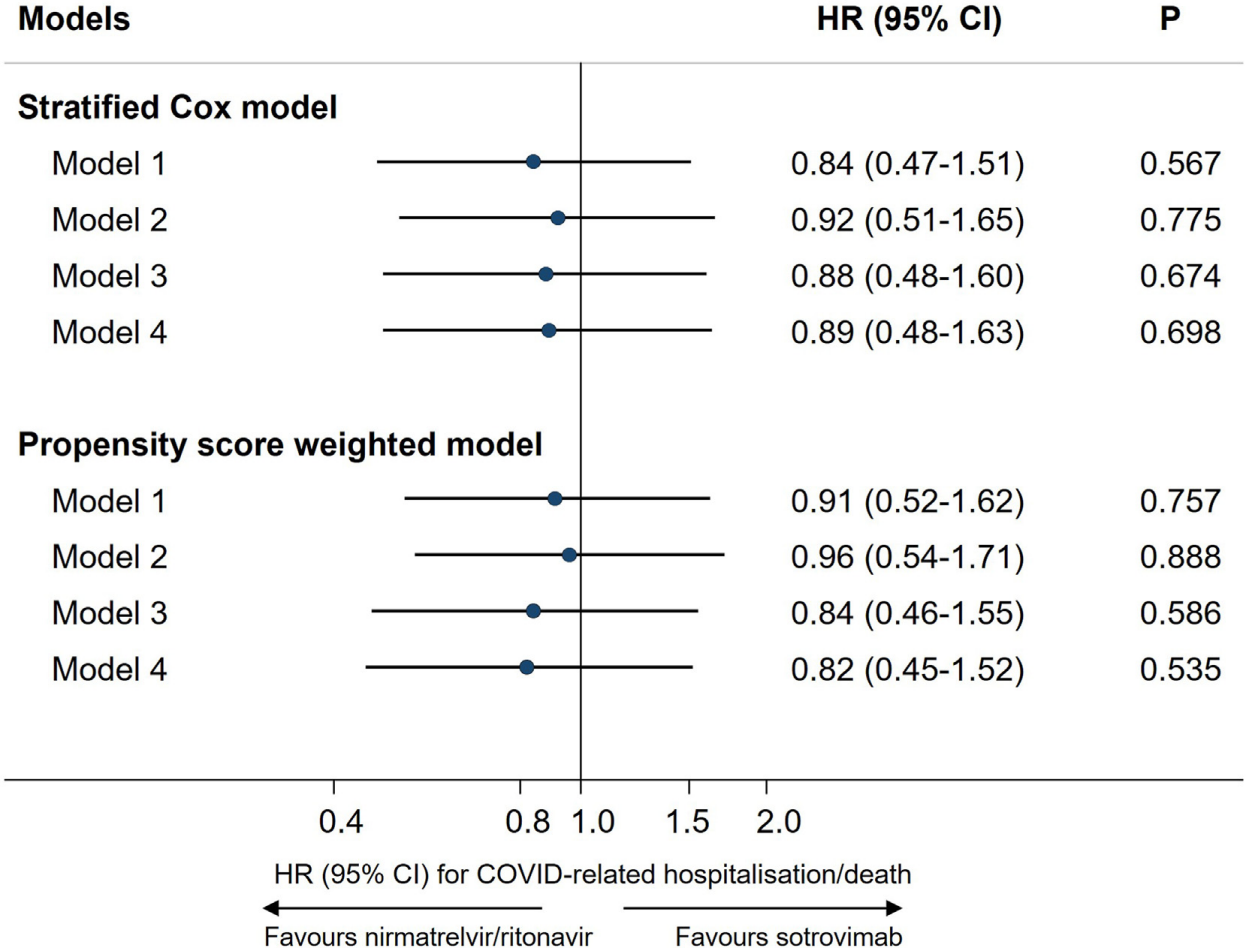
Comparing risk of 28-day COVID-19 related hospitalisation/death between nirmatrelvir/ritonavir versus sotrovimab.

Results of stratified Cox regression showed that, after adjusting for demographic characteristics, high-risk cohort categories, vaccination status, calendar date, BMI category and other comorbidities, treatment with nirmatrelvir/ritonavir was associated with a similar risk of 28-day COVID-19 related hospitalisation/death as treatment with sotrovimab (N = 9026; hazard ratio, HR = 0.89, 95% CI: 0.48–1.63; P = 0.698, with sotrovimab as reference group). Results from propensity score weighted Cox model also showed a non-significant difference between these two treatment groups (Model 4: HR = 0.82, 95% CI: 0.45–1.52; P = 0.535), following confirmation of successful balance of baseline covariates between groups in the weighted sample (SMDs < 0.10, Supplementary Fig. S3A). The HRs remained close to 1 during the sequential covariate adjustment process (ranging from 0.82 to 0.96 across different models; Fig. 2). No violation of the proportional hazards assumption was detected in any model (P > 0.10).

For the secondary outcomes, the analysis of 60-day COVID-19 related hospitalisations/deaths also showed no evidence of difference between the treatment groups (HRs ranging from 1.04 to 1.20 across models; P > 0.05; Table 2). For all-cause hospitalisations/deaths, 240 cases (2.66%) were observed during the 28 days of follow-up after treatment initiation (132 [2.32%] in the nirmatrelvir/ritonavir group and 108 [3.25%] in the sotrovimab group); results of fully-adjusted Cox regression showed weak evidence of lower risk among patients treated with nirmatrelvir/ritonavir (Model 4: HR = 0.78, 95% CI: 0.59–1.03; P = 0.076; Table 2).

**Table 2:** Comparing risks of secondary outcomes between nirmatrelvir/ritonavir versus sotrovimab.

Outcomes	N/Events	Stratified Cox model		PSW analysis	
		HR (95% CI) for nirmatrelvir/ritonavir	P	HR (95% CI) for nirmatrelvir/ritonavir	P
28-day All-cause hospitalisation/death	9015/240				
Model 1		0.69 (0.52–0.90)	0.007	0.66 (0.49–0.88)	0.005
Model 2		0.79 (0.60–1.03)	0.082	0.77 (0.57–1.02)	0.068
Model 3		0.75 (0.57–1.00)	0.047	0.73 (0.54–1.00)	0.051
Model 4		0.78 (0.59–1.03)	0.076	0.74 (0.54–1.01)	0.060
60-day COVID-19 related hospitalisation/death^a^	8794/68				
Model 1		1.05 (0.61–1.79)	0.867	1.14 (0.67–1.93)	0.627
Model 2		1.15 (0.67–1.97)	0.607	1.20 (0.70–2.05)	0.505
Model 3		1.15 (0.66–1.99)	0.629	1.05 (0.59–1.84)	0.877
Model 4		1.18 (0.68–2.06)	0.561	1.04 (0.59–1.85)	0.880

Note: PSW = propensity score weighting; HR = hazard ratio; CI = confidence interval.

Model 1 adjusted for age and sex; Model 2 additionally adjusted for high risk cohort categories; Model 3 further adjusted for ethnicity, IMD quintiles, vaccination status, calendar date; and Model 4 additionally adjusted for BMI category, diabetes, hypertension, chronic cardiac and respiratory diseases.

a For the analysis of 60-day outcome, patients treated after November 1, 2022 were excluded to avoid missing outcome events, as the latest hospitalisation events recorded in our data extraction were on December 30, 2022.

Results of sensitivity analyses were generally consistent with the main findings (Supplementary Table S1). No substantial effect modification was observed for the tested covariates (all P for interaction > 0.05; Supplementary Fig. S4).

### Exploratory analysis comparing nirmatrelvir/ritonavir with molnupiravir

During the study period, 1041 eligible non-hospitalised COVID-19 patients were treated with molnupiravir, with 13 cases (1.25%) of COVID-19 related hospitalisations/deaths observed within the 28 days follow-up (≤5 deaths). Compared to the nirmatrelvir/ritonavir group, the molnupiravir group was older (55.6 versus 52.1 years), had a higher proportion of Down syndrome, immune-mediated inflammatory disorders, diabetes, hypertension, chronic heart diseases and chronic respiratory diseases, and a lower proportion of rare neurological conditions (Supplementary Table S2). There were also some geographic variations in the prescription of these two medications and greater use of molnupiravir earlier during the study period (Supplementary Fig. S1).

Results of stratified Cox regression showed that nirmatrelvir/ritonavir was associated with a significantly lower risk of 28-day COVID-19 related hospitalisation/death compared with molnupiravir (Model 4: HR = 0.45, 95% CI: 0.22–0.94; P = 0.033). Propensity score weighted Cox model yielded a stronger effect estimate favouring nirmatrelvir/ritonavir (Model 4: HR = 0.30, 95% CI: 0.12–0.71; P = 0.007; Table 3, Supplementary Fig. S3B). To explore this discrepancy we inspected the propensity scores and found a smaller range of overlap between propensity score distributions than in the nirmatrelvir/ritonavir versus sotrovimab analysis (Supplementary Fig. S5). Further analysis with trimming^14^ showed that, after excluding both nirmatrelvir/ritonavir and molnupiravir users below the 5th percentile of PS in nirmatrelvir/ritonavir group and above the 95th percentile of PS in molnupiravir, the effect estimate was close to that in the stratified Cox model (N = 3512, HR = 0.52, 95% CI: 0.21–1.28). A comparison of baseline characteristics between those with extremely high PS (mostly nirmatrelvir/ritonavir users) and extremely low PS (mostly molnupiravir users) revealed large differences in age, calendar date of treatment initiation, region and comorbidities (Supplementary Table S3), which were consistent with (but more exaggerated than) the comparison results between all nirmatrelvir/ritonavir users and molnupiravir users (Supplementary Table S2).

**Table 3:** Comparing risks of outcome events between nirmatrelvir/ritonavir versus molnupiravir.

Outcomes	N/Events	Stratified Cox model		PSW analysis	
		HR (95% CI) for nirmatrelvir/ritonavir	P	HR (95% CI) for nirmatrelvir/ritonavir	P
28-day COVID-19 hospitalisation/death	6745/47				
Model 1		0.45 (0.22–0.93)	0.030	0.33 (0.15–0.75)	0.008
Model 2		0.45 (0.22–0.92)	0.028	0.33 (0.15–0.74)	0.007
Model 3		0.47 (0.23–0.97)	0.041	0.34 (0.15–0.77)	0.010
Model 4		0.45 (0.22–0.94)	0.033	0.30 (0.12–0.71)	0.007
28-day All-cause hospitalisation/death	6736/171				
Model 1		0.58 (0.39–0.87)	0.009	0.62 (0.39–0.99)	0.046
Model 2		0.57 (0.38–0.85)	0.005	0.61 (0.38–0.99)	0.045
Model 3		0.59 (0.39–0.88)	0.010	0.65 (0.40–1.05)	0.076
Model 4		0.61 (0.40–0.91)	0.016	0.61 (0.36–1.04)	0.070

Note: PSW = propensity score weighting; HR = hazard ratio; CI = confidence interval.

Model 1 adjusted for age and sex; Model 2 additionally adjusted for high risk cohort categories; Model 3 further adjusted for ethnicity, IMD quintiles, vaccination status, calendar date; and Model 4 additionally adjusted for BMI category, diabetes, hypertension, chronic cardiac, and respiratory diseases.

Among the molnupiravir users, 39 cases (3.75%) of all-cause hospitalisations/deaths were observed within 28 days of follow-up. Compared with molnupiravir users, nirmatrelvir/ritonavir users had a lower risk of 28-day all-cause hospitalisation/death (HR = 0.61, 95% CI: 0.40–0.91 in the stratified Cox model and 0.61, 95% CI: 0.36–1.04 in the propensity score weighted Cox model; Table 3). A similar effect estimate was observed after propensity score trimming (N = 3506, HR = 0.68, 95% CI: 0.41–1.12).

## Discussion

This is one of the largest observational studies to date on the comparative effectiveness of nirmatrelvir/ritonavir versus alternative antiviral medications, covering the Omicron BA.2, BA.5 and BQ.1-dominant periods in the UK.^15^^,^^16^ Our analysis with granular real-world data did not show strong evidence that nirmatrelvir/ritonavir or sotrovimab is more effective than the other for reducing COVID-19 related hospitalisation/death events. The findings remained robust in propensity score weighting analysis and other sensitivity analyses, including during time periods characterised by different dominant Omicron variants. In contrast, the exploratory analyses with molnupiravir users as the comparison group showed a significantly lower risk of severe COVID-19 outcomes among patients prescribed nirmatrelvir/ritonavir.

The key strengths of the OpenSAFELY platform are the scale, level of detail and completeness of the underlying primary care EHR data and the linkage to multiple COVID-19 relevant national databases with near real-time data update.^7^^,^^17^ We used a range of analytic methods to examine robustness of results, and were able to carry out extensive adjustments for confounding and exclusions for contraindications given the availability of granular multisource real-world data. As well as availability of treatment to the population regardless of ability to pay, the uniqueness of the UK data is that the administration of COVID-19 medications in the community is operated by CMDUs that were launched specifically for COVID-19 treatment, following the national prescription guidance and clear eligibility criteria for treatment.^2^ Therefore, our study population is well-characterised with high-quality exposure data based on treatment records in the central system.

Several limitations need to be considered. Despite the granular data on underlying health status and the clinical equipoise in the treatment criteria for nirmatrelvir/ritonavir and sotrovimab (at least during the BA.2 wave), residual confounding cannot be ruled out, in particular related to severity of COVID-19 symptoms or other unmeasured features (such as level of immunosuppression) that may have influenced clinician’s choice of therapy at assessment. Residual confounding might be why we observed weak evidence of lower risk of hospitalisation/death from any cause among people prescribed nirmatrelvir/ritonavir compared to sotrovimab, and with stronger evidence when compared to molnupiravir; though another possible explanation is that more effective treatment of COVID-19 also reduced incidence of other clinical outcomes. Moreover, our definition of primary outcome may be subject to misclassifications and regional heterogeneity, but is still the most specific indicator of severe COVID-19 outcome from the available data sources. We did not use all-cause hospitalisation/death as the primary outcome because most of the treated patients had immunosuppression or severe diseases, thus had a relatively high “baseline rate” of all-cause hospitalisation. In addition, we used primary care data to detect drugs where use of nirmatrelvir/ritonavir was contraindicated,^12^ and thus could have missed prescriptions outside of the GP record (secondary care/“hospital at home”), leading to misclassification of eligibility to receive nirmatrelvir/ritonavir among sotrovimab and molnupiravir users. Finally, the non-hospitalised high-risk patients included in this study are assumed to be only those who met the eligibility criteria made by NHS England and had no contraindications for nirmatrelvir/ritonavir,^2^ thus limiting further generalisation of our findings to other patient groups.

The randomised clinical trials of these nMAbs and antivirals were conducted during periods where previous variants of SARS-CoV-2 were circulating, and mostly in unvaccinated populations, making their relevance to contemporary situations limited. Comparative trials between different treatments have not been conducted, making clinical decisions about treatment for patients eligible to receive any therapy difficult. Both the EPIC-HR trial^3^ for nirmatrelvir/ritonavir and the COMET-ICE trial^4^ for sotrovimab showed evidence of benefit compared to placebo (relative risk = 0.12 and 0.21, respectively). However, a large-scale pragmatic study of molnupiravir, the UK PANORAMIC trial,^18^ showed that molnupiravir did not reduce risk of hospitalisations/deaths among high-risk vaccinated adults with COVID-19 in the community (25,000 participants, adjusted odds ratio = 1.06, 95% Bayesian credible interval: 0.80–1.40). The PANORAMIC trial for nirmatrelvir/ritonavir versus usual care is still ongoing.

A recent literature review shows that a growing body of real-world evidence supports the efficacy of nirmatrelvir/ritonavir among vaccinated adult patients in the Omicron era,^19^ though most of those observational studies were conducted during the BA.1/BA.2 wave. A large-scale cohort study in Israel showed that among high-risk outpatients with COVID-19 aged ≥ 65 years, nirmatrelvir/ritonavir users had substantially lower risk of hospitalisation due to COVID-19 (adjusted HR = 0.27, 95% CI: 0.15–0.49) and death due to COVID-19 (adjusted HR = 0.21, 95% CI: 0.05–0.82) compared to untreated patients; whereas no association was observed in patients aged <65 years (adjusted HR = 0.74, 95% CI: 0.35–1.58).^5^ A large-scale observational study of non-hospitalised COVID-19 patients in Hong Kong SAR showed that nirmatrelvir/ritonavir use was associated with lower risk of all-cause mortality (HR = 0.34, 95% CI: 0.22–0.52) and hospital admission due to COVID-19 (HR = 0.76, 95% CI: 0.67–0.86) compared with non-use, with no effect modification by age.^6^ In contrast, molnupiravir use was associated with lower risk of death (HR = 0.76, 95% CI: 0.61–0.95) but not hospitalisation (0.98, 95% CI: 0.89–1.06) compared with non-use.^6^ Another two cohort studies in the US population showed that non-hospitalised COVID-19 patients who were prescribed nirmatrelvir/ritonavir had a lower risk of all-cause hospitalisation or death than non-users.^20^^,^^21^ Evidence for the safety of Paxovid in routine clinical use, however, is still limited, and the concerns of viral rebound and recurrence of COVID-19 symptoms following nirmatrelvir/ritonavir treatment need further investigation.^19^^,^^22^

Given the emerging evidence on COVID-19 therapy assessment, including real-world data from OpenSAFELY,^17^ clinical guidelines have been updated over time and discrepancies appeared across different countries and authorities (e.g., the difference between WHO^8^ and NICE guidelines^9^ regarding sotrovimab as mentioned in the Introduction). In the NHS guidance published on February 10, 2022,^2^ nirmatrelvir/ritonavir and sotrovimab were recommended as the first-line treatments and molnupiravir as the third-line option (following remdesivir); on November 27, 2022,^11^ sotrovimab was de-prioritised and only to be considered by exception, while the recommendation level of the other two remained unchanged; however, the latest version of NHS guidance published on May 11, 2023^23^ adds back sotrovimab as the second-line option based on more recent evidence, while nirmatrelvir/ritonavir remaining as the first-line and molnupiravir moved to the fourth-line.

While randomised clinical trials are rightly considered a gold standard for studying drug effectiveness, rapidly evolving SARS-CoV-2 variants can mean results of COVID-19 therapeutics trials may become quickly outdated. Additionally, there remain areas of uncertainty where clinical trials have not been conducted such as comparative effectiveness between different therapeutic agents or for populations underrepresented in clinical trials. In vitro data might provide conclusive evidence of loss of drug effect but where different variants are circulating, or where in vitro data shows intermediate effects, the clinical benefits of treatment in a whole population may be uncertain.^24^ For instance, previous in vitro studies showed that sotrovimab remained active against the Omicron BA.1 variant but exhibited marked reduction in neutralising activity to the Omicron BA.2, BA.4 and BA.5 variants^25^^,^^26^; whereas several recent in vivo and in vitro experiments showed that sotrovimab retains activity (or partial activity) against BQ.1 and XBB variants.^27^, ^28^, ^29^ Therefore, measuring the continued effectiveness of treatments for a rapidly evolving virus presents a uniquely difficult situation for decisions made by healthcare regulators and those providing treatment recommendations. Careful analysis of routinely-collected healthcare data can provide rapid estimates of drug effectiveness and safety within the whole population and can provide critical information which can be used alongside in vitro data to support future decision-making.^17^^,^^30^ On the basis of ongoing availability of data regarding COVID-19 therapeutics, we aim to continue providing real-world pharmacoepidemiologic evidence in the context of the circulating variants. The UK government has committed to improving the health data infrastructure,^31^^,^^32^ we believe this study demonstrates that OpenSAFELY can be used to deliver practical applications of these commitments around real-world evidence.

In conclusion, in routine care of non-hospitalised high-risk adult patients with COVID-19 in England, we observed no substantial difference in the risk of severe COVID-19 outcomes between those who received nirmatrelvir/ritonavir and sotrovimab during Omicron BA.2, BA.5, and BQ.1-dominant periods.

## Contributors

The study was conceptualised by LAT, BZ, IJD, BM, and BG. BZ and ACAG contributed to the coding and statistical analysis and verified the underlying data. BZ and LAT drafted the original version of the manuscript. AM, BG, BM, CB, JP, HJC, and SCJB contributed to information governance and project administration. All authors were involved in critical revisions and approving the final draft for submission. All authors had access to the aggregated data in the OpenSAFELY platform and accept responsibility for the decision to submit for publication.

## Data sharing statement

All data were linked, stored and analysed securely within the OpenSAFELY platform https://opensafely.org/. All code for data management and analysis, as well as codelists, are shared openly for review and re-use under MIT open license (https://github.com/opensafely/Paxlovid-and-sotrovimab). All iterations of the pre-specified study protocol are archived with version control (https://github.com/opensafely/Paxlovid-and-sotrovimab/tree/main/docs). Detailed pseudonymised patient data is potentially re-identifiable and therefore not shared.

## Information governance and ethical approval

NHS England is the data controller; TPP is the data processor; all study authors using OpenSAFELY have the approval of NHS England. This implementation of OpenSAFELY is hosted within the TPP environment which is accredited to the ISO 27001 information security standard and is NHS IG Toolkit compliant; patient data has been pseudonymised for analysis and linkage using industry standard cryptographic hashing techniques; all pseudonymised datasets transmitted for linkage onto OpenSAFELY are encrypted; access to the NHS England OpenSAFELY COVID-19 serviceis via a virtual private network (VPN) connection; the researchers hold contracts with NHS England and only access the platform to initiate database queries and statistical models; all database activity is logged; only aggregate statistical outputs leave the platform environment following best practice for anonymisation of results such as statistical disclosure control for low cell counts. The service adheres to the obligations of the UK General Data Protection Regulation (UK GDPR) and the Data Protection Act 2018. The service previously operated under notices initially issued in February 2020 by the Secretary of State under Regulation 3(4) of the Health Service (Control of Patient Information) Regulations 2002 (COPI Regulations), which required organisations to process confidential patient information for COVID-19 purposes; this set aside the requirement for patient consent. As of 1 July 2023, the Secretary of State has requested that NHS England continue to operate the Service under the COVID-19 Directions 2020. In some cases of data sharing, the common law duty of confidence is met using, for example, patient consent or support from the Health Research Authority Confidentiality Advisory Group. Taken together, these provide the legal bases to link patient datasets using the service. GP practices, which provide access to the primary care data, are required to share relevant health information to support the public health response to the pandemic, and have been informed of how the service operates.

This study was approved by the Health Research Authority (REC reference 20/LO/0651) and by the LSHTM Ethics Board (reference 21863).

## Declaration of interests

BG has received research funding from the Laura and John Arnold Foundation, the NHS National Institute for Health Research (NIHR), the NIHR School of Primary Care Research, NHS England, the NIHR Oxford Biomedical Research Centre, the Mohn-Westlake Foundation, NIHR Applied Research Collaboration Oxford and Thames Valley, the Wellcome Trust, the Good Thinking Foundation, Health Data Research UK, the Health Foundation, the World Health Organisation, UKRI MRC, Asthma UK, the British Lung Foundation, and the Longitudinal Health and Wellbeing strand of the National Core Studies programme; he is a Non-Executive Director at NHS Digital; he also receives personal income from speaking and writing for lay audiences on the misuse of science. BMK is also employed by NHS England working on medicines policy and clinical lead for primary care medicines data. AM is a member of RCGP health informatics group and the NHS Digital GP data Professional Advisory Group, and received consulting fee from Induction Healthcare. SJWE was paid for attendance at meetings of Coalition for Epidemic Preparedness Innovations (CEPI) Meta–Data Safety Monitoring Board. IJD has received research grants from GSK and AstraZeneca and holds shares in GSK. LAT has received research funding from MRC, Wellcome, NIHR and GSK, consulted for Bayer in relation to an observational study of chronic kidney disease (unpaid), and is a member of 4 non-industry funded (NIHR/MRC) trial advisory committees (unpaid) and MHRA Expert advisory group (Women’s Health). ELH received NIHR post doctoral fellowship. REC has shares in AstraZeneca. VM received grant from NIHR. VS received speaker fees from Bayer AG. JP is employed by TPP SystmOne in the role of Clinical Director. RME received research grants from NIHR, HDR UK, IMI2, New Ventures Fund. JT received AstraZeneca grant for unrelated COVID-19 research. Other authors declared no conflict of interest.
