# Supplementary material for: Comparative effectiveness of nirmatrelvir/ritonavir versus sotrovimab and molnupiravir for preventing severe COVID-19 outcomes in non-hospitalised high-risk patients during Omicron waves: observational cohort study using the OpenSAFELY platform
Source: Lancet Reg Health Eur. 2023 Oct 8;34:100741. doi: 10.1016/j.lanepe.2023.100741 (PMC10624988; doi:10.1016/j.lanepe.2023.100741)
Supplement: Supplementary Figs. S1–S5 and Tables S1–S3 [file mmc1.docx]

# **Supplement**


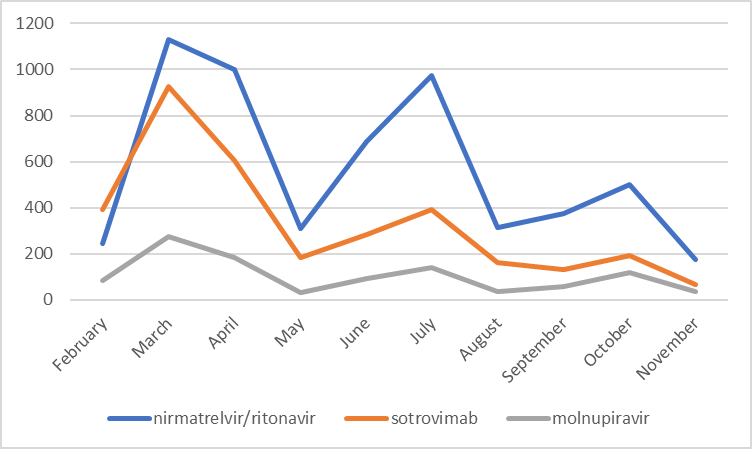


**Supplementary Figure 1. Monthly counts of prescriptions of nirmatrelvir/ritonavir, sotrovimab and molnupiravir between February and November, 2022.**

Note: All counts were rounded to the nearest 5 in compliance with re-identification minimisation requirements in OpenSAFELY.

**
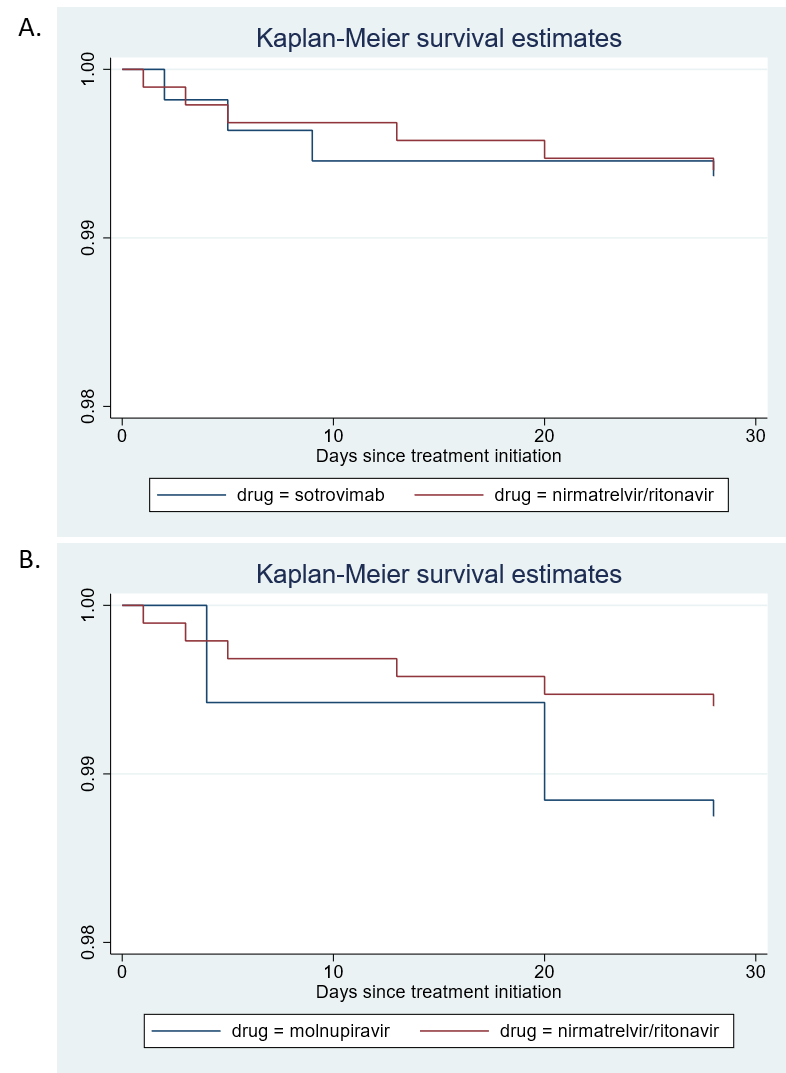
**

**Supplementary Figure 2. Kaplan-Meier curves of COVID-19 related hospitalisation/death in patients receiving nirmatrelvir/ritonavir vs. sotrovimab (A) and nirmatrelvir/ritonavir vs. molnupiravir (B).**

Note: Kaplan–Meier steps are delayed until >5 events occur in compliance with re-identification minimisation requirements in OpenSAFELY.

**
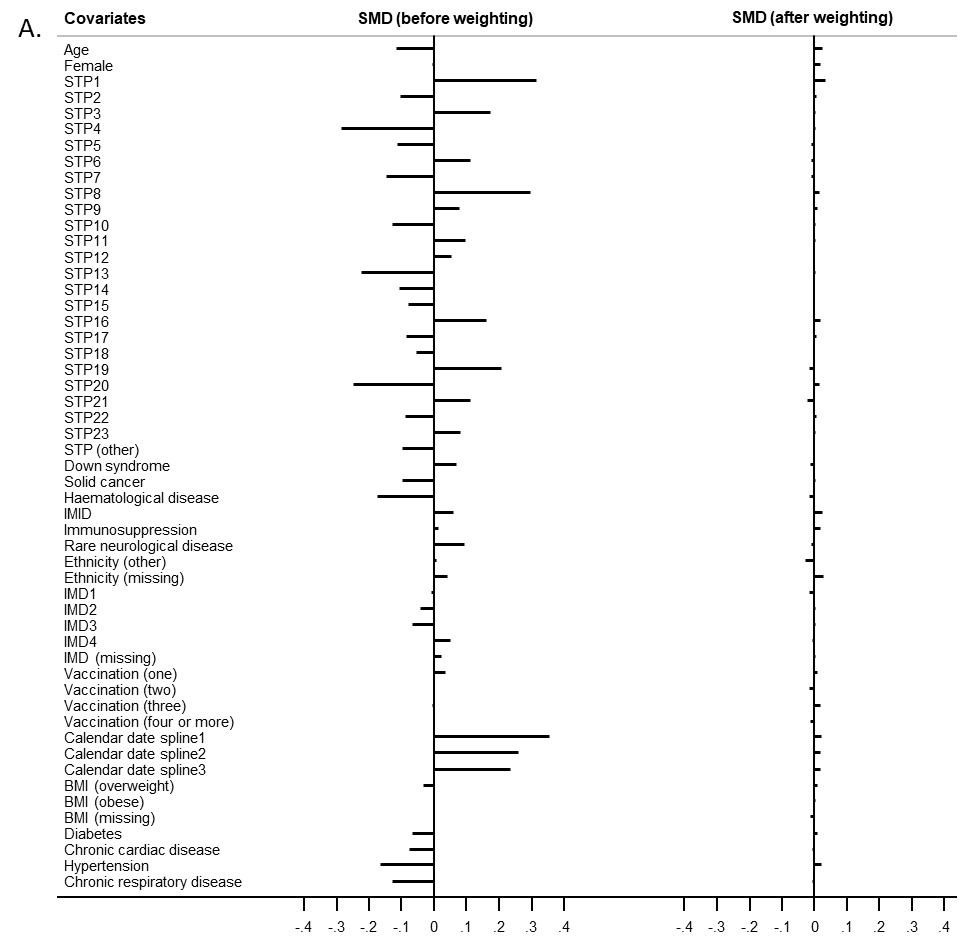
**

**
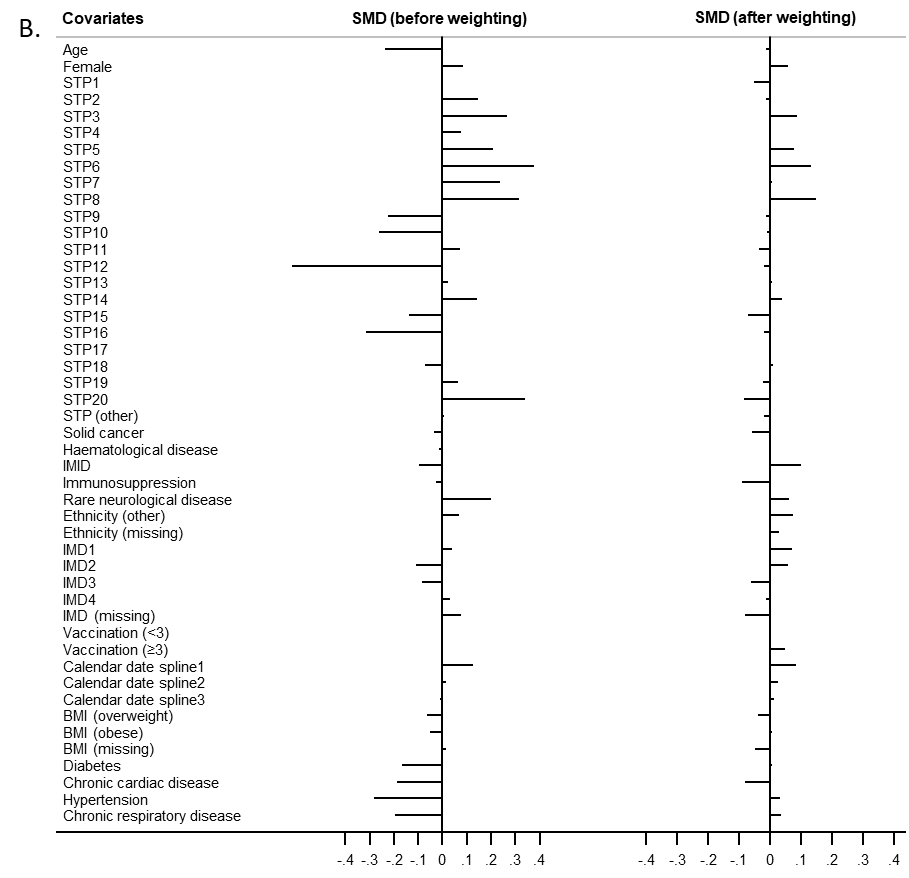
**

**Supplementary Figure 3. Balance check of baseline covariates before and after propensity score weighting between nirmatrelvir/ritonavir vs. sotrovimab (A) and nirmatrelvir/ritonavir vs. molnupiravir (B).**

Note: SMD=standardised mean difference; STP=Sustainability Transformation Partnerships; IMID=Immune-Mediated Inflammatory Diseases; IMD=Index of Multiple Deprivation; BMI=body mass index.

**Supplementary Table 1. Sensitivity analysis of 28-day COVID-19 related hospitalisation/death between nirmatrelvir/ritonavir vs. sotrovimab.**

| **Sensitivity analysis** | **N** | **Events** | **HR (95% CI) for nirmatrelvir/ritonavir (ref=sotrovimab)** | **P** |
| --- | --- | --- | --- | --- |
| complete-case analysis | 7811 | 47 | 0.92 (0.47-1.77) | 0.791 |
| multiple imputation for covariates | 9026 | 55 | 0.89 (0.48-1.63) | 0.700 |
| additionally adjusting for days between test positive and treatment initiation, and months between last vaccination date and treatment initiation | 9026 | 55 | 0.96 (0.52-1.79) | 0.905 |
| additionally adjusting for rural-urban, comorbidity, housebound | 9026 | 55 | 0.92 (0.50-1.69) | 0.778 |
| using restricted cubic splines for age | 9026 | 55 | 0.89 (0.48-1.64) | 0.711 |
| excluding patients with treatment records of both sotrovimab and molnupiravir, or with treatment records of any other therapies | 8987 | redacted | 0.89 (0.48-1.63) | 0.703 |
| excluding patients who initiated treatment after 5 days since positive SARS-CoV-2 test | 8968 | redacted | 0.87 (0.47-1.60) | 0.658 |
| adding back patients who did not have a positive SARS-CoV-2 test record before treatment or initiated treatment after 7 days since positive SARS-CoV-2 test | 10376 | 74 | 1.11 (0.65-1.90) | 0.701 |
| adding back patients who had missing high-risk cohort information | 13276 | 69 | 1.04 (0.60-1.81) | 0.893 |
| creating a 1-day lag in the follow-up start date | 8994 | 43 | 1.00 (0.50-2.00) | 0.995 |
| creating a 2-day lag in the follow-up start date | 8980 | 39 | 0.92 (0.45-1.90) | 0.826 |
| excluding those with medications in “Drugs consider risks and benefits” codelist for nirmatrelvir/ritonavir [11] within 180 days before baseline | 6692 | 42 | 1.23 (0.59-2.56) | 0.572 |
| not excluding contraindications, adjusting for them as covariates | 15705 | 145 | 0.73 (0.48-1.11) | 0.139 |
| additionally adjusting for vaccine type for the latest COVID-19 vaccination | 9026 | 55 | 0.88 (0.47-1.67) | 0.704 |
| restricting to patients receiving BNT vaccine in the latest COVID-19 vaccination | 6228 | 38 | 0.83 (0.40-1.70) | 0.602 |
| restricting to patients receiving Moderna vaccine in the latest COVID-19 vaccination | 2103 | 9 | 1.05 (0.16-6.90) | 0.952 |
| extending to 3-month follow-up | 9026 | 74 | 1.14 (0.68-1.93) | 0.620 |

**Supplementary Table 2. Baseline characteristics of patients receiving nirmatrelvir/ritonavir or molnupiravir.**

| **Characteristics** | **nirmatrelvir/ritonavir group** | **Molnupiravir group** |
| --- | --- | --- |
| N | 5704 | 1041 |
| Age (year), mean (SD) | 52.1 (14.7) | 55.6 (15.5) |
| Female, n (%) | 3830 (67.2) | 658 (63.2) |
| White, n (%) | 5245 (93.5) | 974 (95.1) |
| IMD quintile, n (%) |  |  |
| 1 (most deprived) | 585 (10.6) | 95 (9.3) |
| 2 | 860 (15.6) | 200 (19.6) |
| 3 | 1240 (22.4) | 264 (25.8) |
| 4 | 1401 (25.3) | 241 (23.6) |
| 5 (least deprived) | 1445 (26.1) | 222 (21.7) |
| Region (NHS), n (%) |  |  |
| East | 1456 (25.5) | 505 (48.5) |
| London | 270 (4.7) | 20 (1.9) |
| East Midlands | 1345 (23.6) | 43 (4.1) |
| West Midlands | 71 (1.2) | 10 (1.0) |
| North East | 144 (2.5) | 7 (0.7) |
| North West | 545 (9.6) | 21 (2.0) |
| South East | 542 (9.5) | 128 (12.3) |
| South West | 593 (10.4) | 204 (19.6) |
| Yorkshire | 738 (12.9) | 103 (9.9) |
| High risk cohorts, n (%) |  |  |
| Down syndrome | 244 (4.3) | 62 (6.0) |
| Solid cancer | 615 (10.8) | 124 (11.9) |
| Haematological disease | 717 (12.6) | 135 (13.0) |
| Immune-mediated inflammatory diseases | 2370 (41.6) | 481 (46.2) |
| Immunosuppression | 610 (10.7) | 120 (11.5) |
| HIV/AIDS | 17 (0.3) | 7 (0.7) |
| Rare neurological disease | 1595 (28.0) | 203 (19.5) |
| BMI (kg/m^2^), mean (SD) | 28.3 (6.7) | 28.9 (6.8) |
| Comorbidities, n (%) |  |  |
| Diabetes | 689 (12.1) | 187 (18.0) |
| Chronic cardiac disease | 309 (5.4) | 109 (10.5) |
| Hypertension | 1274 (22.3) | 363 (34.9) |
| Chronic respiratory disease | 894 (15.7) | 243 (23.3) |
| Vaccination status, n (%) |  |  |
| None | 81 (1.4) | 19 (1.8) |
| One vaccination | 77 (1.4) | 7 (0.7) |
| Two vaccinations | 223 (3.9) | 40 (3.8) |
| Three vaccinations | 2782 (48.8) | 509 (48.9) |
| Four or more | 2541 (44.6) | 466 (44.8) |
| Days between test positive and treatment, median (IQR) | 1 (1-2) | 2 (1-2) |
| Weeks between campaign start and treatment, median (IQR) | 26 (16-32) | 19 (14-32) |

**Supplementary Table 3. Baseline characteristics of patients with extreme propensity scores (PS) in the nirmatrelvir/ritonavir vs. molnupiravir analysis.**

| **Characteristics** | **Extremely low PS** | **Extremely high PS** |
| --- | --- | --- |
| N | 669 | 2564 |
| Age (year), mean (SD) | 57.6 (15.2) | 50.2 (14.7) |
| Female, n (%) | 413 (61.7) | 1779 (69.4) |
| White, n (%) | 629 (95.5) | 2337 (92.3) |
| IMD quintile, n (%) |  |  |
| 1 (most deprived) | 57 (8.7) | 267 (10.9) |
| 2 | 155 (23.6) | 342 (13.9) |
| 3 | 170 (25.8) | 476 (19.4) |
| 4 | 139 (21.1) | 652 (26.5) |
| 5 (least deprived) | 137 (20.8) | 720 (29.3) |
| Region (NHS), n (%) |  |  |
| East | 461 (68.9) | 445 (17.4) |
| London | ≤5 | 120 (4.7) |
| Midlands | ≤5 | 1170 (45.6) |
| North East | ≤5 | 120 (4.7) |
| North West | ≤5 | 478 (18.6) |
| South East | 41 (6.1) | 41 (1.6) |
| South West | 153 (22.9) | 22 (0.9) |
| Yorkshire | redacted | 168 (6.6) |
| High risk cohorts, n (%) |  |  |
| Down syndrome | 45 (6.7) | 106 (4.1) |
| Solid cancer | 70 (10.5) | 220 (8.6) |
| Haematological disease | 89 (13.3) | 293 (11.4) |
| Immune-mediated inflammatory diseases | 318 (47.5) | 1003 (39.1) |
| Immunosuppression | 75 (11.2) | 237 (9.2) |
| Rare neurological disease | 117 (17.5) | 886 (34.6) |
| BMI (kg/m^2^), mean (SD) | 29.1 (6.3) | 28.3 (6.9) |
| Comorbidities, n (%) |  |  |
| Diabetes | 149 (22.3) | 253 (9.9) |
| Chronic cardiac disease | 102 (15.3) | 107 (4.2) |
| Hypertension | 290 (43.4) | 445 (17.4) |
| Chronic respiratory disease | 201 (30.0) | 340 (13.3) |
| Vaccination status, n (%) |  |  |
| None | 14 (2.1) | 34 (1.3) |
| One vaccination | 6 (9.0) | 34 (1.3) |
| Two vaccinations | 17 (2.5) | 103 (4.0) |
| Three vaccinations | 350 (52.3) | 1269 (49.5) |
| Four or more | 282 (42.2) | 1124 (43.8) |
| Days between test positive and treatment, median (IQR) | 1 (1-2) | 2 (1-2) |
| Weeks between campaign start and treatment, median (IQR) | 17 (13-31) | 28 (17-32) |


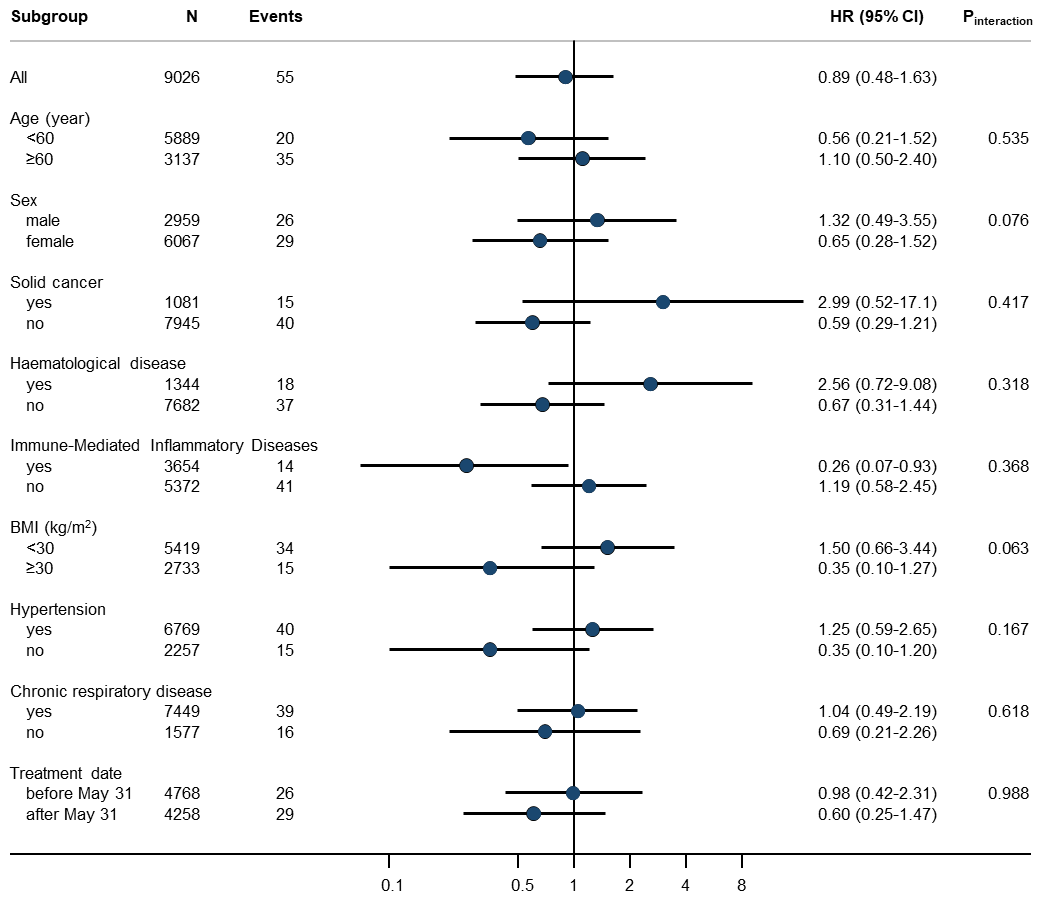


**Supplementary Figure 4. Subgroup analysis of nirmatrelvir/ritonavir vs. sotrovimab in association with risk of 28-day COVID-19 related hospitalisation/death.**

Note: HR=hazard ratio; CI=confidence interval; BMI=body mass index. Subgroup analyses were based on the fully-adjusted stratified Cox model (Model 4). P for interaction between drug group and each of the following variables was: rare neurological conditions (0.078), diabetes (0.046), chronic cardiac diseases (0.723), days between test positive and treatment initiation (0.367), and White ethnicity (0.956); no analyses within each level of these variables were done because of lack of sample size or outcome events within the subset of population.

**
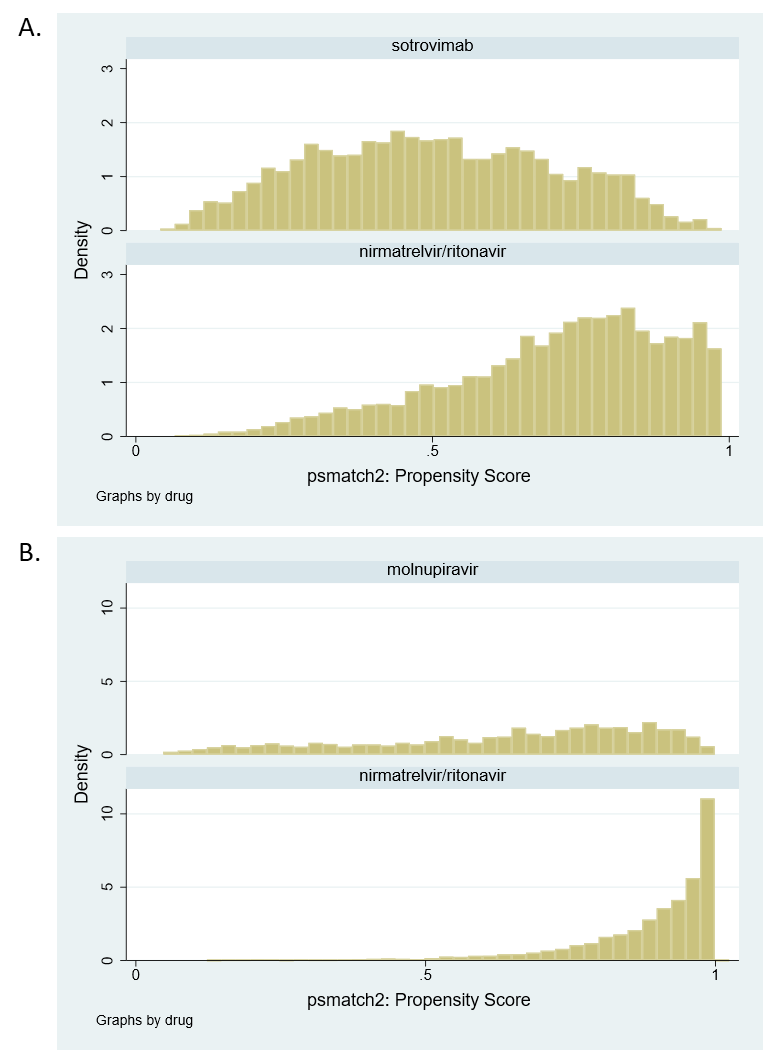
**

**Supplementary Figure 5. Histograms of propensity scores in nirmatrelvir/ritonavir vs. sotrovimab (A) and nirmatrelvir/ritonavir vs. molnupiravir analyses (B).**
